# Defending the Democratic Argument for Limitarianism: A Reply to Volacu and Dumitru

**DOI:** 10.1007/s11406-018-0030-6

**Published:** 2018-10-25

**Authors:** Dick Timmer

**Affiliations:** Department of Philosophy and Religious Studies, Utrecht University, Janskerkhof 13, 3512 BL Utrecht, The Netherlands

**Keywords:** Political philosophy, Limitarianism, Distributive justice, Political equality, Taxation

## Abstract

In this paper, I argue that limitarian policies are a good means to further political equality. Limitarianism, which is a view coined and defended by Robeyns (2017), is a partial view in distributive justice which claims that under non-ideal circumstances it is morally impermissible to be rich. In a recent paper, Volacu and Dumitru (2018) level two arguments against Robeyns’ Democratic Argument for limitarianism. The Democratic Argument states that limitarianism is called for given the undermining influence current inequalities in income and wealth have for the value of democracy and political equality. Volacu and Dumitru’s Incentive Objection holds that limitarianism places an excessive and inefficient burden on the rich in ensuring political equality. The Efficacy Objection holds that even if limitarianism limits excessive wealth it still fails to ensure the preservation of political equality. In this paper, I will argue that both of these objections fail, but on separate grounds. I argue that the Incentive objection fails because one could appeal to limitarian policies that are different from the ones discussed by Volacu and Dumitru and which escape the problem of reduced productivity. I argue against the Efficacy Objection that limitarian policies are a partial but highly valuable step towards establishing political equality, and that they can and should complement or be complemented by other strategies.

## Introduction

Limitarianism is a partial view in distributive justice which claims that under non-ideal circumstances it is morally impermissible to be rich (Robeyns 2017, p. 1).^1^ In a recent paper, Volacu and Dumitru (2018) level two arguments against Robeyns’ *Democratic Argument* for limitarianism, which I will label the *Incentive Objection* and the *Efficacy Objection*. In this paper, I will argue that both of these objections fail, but on separate grounds.

The Democratic Argument states that limitarianism is called for given the undermining influence current income and wealth inequalities have on democracy and the value of political equality (cf. Robeyns 2017, pp. 6–10; Volacu and Dumitru 2018, pp. 4, 5, 6–10). More precisely, the argument runs as follows: political equality requires that nobody should have more or less political influence just because they are richer. However, excessive wealth allows the rich more political influence precisely because they are richer. This means that excessive wealth threatens political equality. Therefore, to the extent that limitarianism limits excessive wealth, limitarianism is pro tanto justified as a means to promote political equality.

Volacu and Dumitru argue that the Democratic Argument proposes what they call *strong limitarianism* rather than *weak limitarianism*, which means that the argument “advocates taxing any income above the riches line […] at 100%,” rather than the ‘weaker’ policy of seeking “to enact revenue-maximising tax policies” (Volacu and Dumitru 2018, pp. 5, 13).^2^ In light of that, the Incentive Objection against the Democratic Argument holds that strong limitarianism places an excessive and inefficient burden on the rich in ensuring political equality. The Efficacy Objection holds that even if limitarianism limits excessive wealth it still fails to ensure the preservation of political equality. In what follows, I will first distinguish distributive limitarianism and policy limitarianism as two interpretations of Robeyns’ limitarian doctrine, and elaborate on some conceptual issues regarding policy limitarianism and limitarian policies (Section 2). I will then argue that the Incentive objection fails because one could appeal to limitarian policies that are different from the ones discussed by Volacu and Dumitru and which escape the problem of reduced productivity (Section 3). I argue against the Efficacy Objection that limitarian policies are a partial but highly valuable step towards establishing political equality, and that they can and should be complemented with other strategies (Section 4).

## Distributive Limitarianism and Policy Limitarianism

In her article, Robeyns sets limitarianism on a par with pattern-sensitive distributive principles such as egalitarianism, sufficientarianism, and prioritarianism (Robeyns 2017, p. 1). I will call this *distributive limitarianism*. According to distributive limitarianism, a fair distribution of the metric of justice, e.g. primary goods or capabilities, requires that no-one has more than a certain amount of that metric. However, the explicit focus on wealth and income of limitarianism suggests a second interpretation, which we can label *policy limitarianism.* Policy limitarianism holds that policies which limit the amount of income or wealth an individual can possess constitute the normatively appropriate steps to bring about a more just state of affairs.^3^ Though neither Robeyns nor Volacu and Dumitru make the distinction between distributive limitarianism and policy limitarianism, it is clear from at least Volacu and Dumitru’s arguments that they have limitarian policies rather than limitarian distributive principles in mind when assessing limitarianism (e.g. Volacu and Dumitru 2018, p. 5, 7, 10–11). However, if limitarianism is indeed about policies rather than principles, limitarians have more policies at their disposal than weak limitarianism and strong limitarianism, or so I will argue.

As I understand it, policy limitarianism is a non-ideal and partial view on how to instantiate and promote certain values, such as political equality, equal social status, or mental wellbeing. The non-ideality refers to the fact that limitarian policies are supposed to limit excessive wealth to promote those values in light of the actual distribution of income and wealth. Policy limitarianism does not rely on the claim that wealth is morally wrong in itself. The claim is rather that there are benefits to focusing on excessive wealth to promote political equality. Under non-ideal circumstances policies which tackle inequality rather than riches may not be enough to fight the distortion of excessive wealth on political equality among members of a society. For example because of the still and rapidly increasing accumulation of wealth by the so-called 1% despite the fact that many redistributive measures have already been taken by democratic governments (Piketty 2013). Hence, even if the underlying problem is inequality in political power resulting from inequalities in wealth, we have reasons to explore measures which directly target excessive wealth. Whether or not such measures are efficient and effective is something to which I will turn later on.

Limitarian policies, then, are justified by referring to convictions about moral values and principles, and by empirical facts about which policies (if any) will actually instantiate, promote or otherwise deliver on those values and principles. Partiality refers to the fact that limitarian policies are not *sufficient* to establish political equality. They may not even be *necessary* to establish political equality, depending on what one understands the non-ideal circumstances to be precisely. Below, however, I will argue that limitarian policies are one of the most promising strategies to combat political inequality for a variety of reasons. I will elaborate on these reasons when responding to the Incentive Objection and the Efficacy Objection.

Before doing so, let me briefly comment on what makes a policy ‘limitarian’, because this will play an important role in the response to both objections. A policy can be limitarian in structure or in aim (or both). A policy is limitarian in structure if it directly targets the amount of wealth individuals can appropriate, e.g. a maximum wage or a tax on capital income. The reason to employ such policies need not be a concern for the excessively wealthy as such. One may also draw on limitarian policies to, for example, ensure just or fair wages or to fight unfair advantages due to natural luck; or, indeed, to protect political equality. A policy is limitarian in aim if its goal is to establish social structures that will tend not to bring about excessive individual wealth. A policy is limitarian both in structure and in aim if it establishes social structures that will tend not to bring about excessive individual wealth by directly targeting the amount of wealth individuals can appropriate.

We can further refine the concept of limitarian policies by adding the notions of redistribution and predistribution.^4^ Redistributive limitarian policies limit excessive wealth for the purpose of reallocating the benefits of social cooperation through tax-and-transfer policies. Both weak limitarianism and strong limitarianism are variations on such redistributive limitarian policies. Predistributive limitarian policies aim to limit excessive wealth via manipulation of initial holdings in order to generate a fair distribution of opportunities, for example via inheritance tax (cf. Dietsch 2010). I will turn to this distinction below in response to the Incentive Objection.

## The Incentive Objection

The Incentive Objection asserts that strong limitarianism disincentivizes the rich to be maximally productive, and that we have moral reasons to avert that. Hence, even if limitarian policies would in fact establish political equality, there are overriding reasons to shy away from using such measures. Volacu and Dumitru (2018, p. 8), for example, say that strong limitarianism “would make it harder to move towards the goal of meeting [unmet urgent needs], since the fiscal policy it prescribes would not be revenue-maximizing.” And even if all urgent needs are met and political equality is established, strong limitarianism may still have significant harmful effects. This is because the benevolent rich do much more to establish justice, such as engaging “in activities associated with combating climate change, [or contributing] to the establishment and development of democracy-building non-governmental organisations in countries that are in a process of transition from autocratic or totalitarian regimes to democratic ones” (Volacu and Dumitru 2018, p. 8). However, if strong limitarianism is adopted, Volacu and Dumitru argue, “instead of having an ample amount of surplus money which they could donate to these causes, the benevolent rich would have to relinquish all of it” (Volacu and Dumitru 2018, p. 8).

Though I will argue that the Incentive Objection ultimately fails to refute the Democratic Argument, it rightly stresses that policies which incentivize people to be maximally productive may threaten political equality, and policies which promote political equality may disincentivize individuals. In fact, all policies, including limitarian ones, are trade-offs between different values. But even if limitarianism has negative effects on the incentives of the rich (which is, in the end, an empirical question), that does not mean that limitarianism must be abandoned. That choice ultimately depends on the trade-offs one is willing to make, e.g. between economic growth, wellbeing, and political equality. However, and to respond to the Incentive Objection more directly, the trade-off between political equality and productivity need not be as serious as Volacu and Dumitru suggest. This is partly because they underestimate the kinds of policies that are at a limitarian’s disposal, and partly because they overestimate the good that is done by the rich. Let me now turn to these claims in turn.

First, Volacu and Dumitru’s combination of the Democratic Argument with strong limitarianism excludes a broad array of possible and arguably much more attractive limitarian policies that promote political equality. To illustrate, recall the distinction between redistributive limitarianism and predistributive limitarianism. There are many variations in redistributive and predistributive policies, and they have different effects on incentives. Volacu and Dumitru, however, give only two very specific limitarian policies. Another limitarian policy would be a high inheritance tax. Some philosophers and economists argue that an inheritance tax, which could be limitarian both in structure and in aim, disincentivizes people to be maximally productive (e.g. Haslett 1986; McCaffery 2000). Such an objection to a ‘limitarian’ inheritance tax echoes Volacu and Dumitru’s concern for the levelling down tendency of strong limitarianism. However, one may question whether the effects of an inheritance on productivity are indeed that severe (cf. Conesa et al. 2009; Piketty and Saez 2013).

More importantly, there may be alternative taxation schemes that are fairer and but still revenue-maximizing. The central claim about why the Incentive Objection fails rests on the possibility of predistributive limitarian policies which target the accumulation of wealth without being disincentivizing and bad for productivity. Though this requires extensive empirical research possible examples of such schemes may be found in Meade (2012) and Halliday (2018). Let me discuss Halliday’s interpretation of the Rignano scheme as an example. The core feature of the Rignano scheme is that it makes inheritance tax sensitive not to the monetary value of the inheritance alone but also to its age (cf. Rignano 1925, p. 37–38). This means that individuals are incentivized to be productive, because they can—depending on the details of the scheme—only bequeath (part of) the wealth they acquire if it is more than they received from their parents. This has two important benefits over common inheritance tax schemes which mainly target the amount of wealth transferred rather than its age. Though it is difficult to pin down exactly what makes people want to bequeath wealth, one often evoked explanation is their wish to leave something to their children or relatives. If this is true, the Incentive Objection would suggest that without the option to bequeath, one takes away an important incentive for people to be maximally productive. Being soft on first-generation transfers, as proposed by the Rignano scheme, incentivizes people to be productive because the more productive they are, the more they will be able to give. This takes away the main worry raised by the Incentive Objection. Furthermore, being increasingly strict on second-, third- and nth-generation transfers helps to curb the accumulation of wealth and prevents economic segregation over time.^5^ This, in turn, ties in with the main rationale behind the Democratic Argument for limitarianism.

Hence, the first response to the Incentive Objection is that the trade-off between political equality and productivity via limitarian policies need not be as stark as Volacu and Dumitru suggest. There are other, and arguably better, ways to slow down or stop the accumulation of excessive wealth whilst encouraging individuals to be productive. Furthermore, it is important to bear in mind that limitarian policies are only a partial means to promote political equality. Perhaps that when assessed as an isolated strategy strong limitarianism would indeed be the most effective means to promote political equality, but if we compare it to and view it in relation with other policies (e.g. weak limitarianism) strong limitarianism loses much of its initial appeal.

The second response to the Incentive Objection stresses that apart from underestimating the range of limitarian policies, Volacu and Dumitru overestimate the good that is done by the rich and by allowing extreme wealth. To start with, Volacu and Dumitru hold that the benevolent rich further justice, meet unmet urgent needs and establish other kinds of goods (Volacu and Dumitru 2018, p. 8). However, it is far from evident that the benevolent rich are fair and just distributive agents, as Dumitru and Volacu suggest (cf. Gomberg 2002; Slim 2002). This ties in with the issues about cognitive biases and economic inequality that we will turn to below, as well as with the question how much good is *in fact* being done by the benevolent rich. However, if the benevolent rich are fair agents of justice, limitarian policies need not be detrimental to philanthropy either. For example, not excessively rich but still affluent people could pool their resources and donate it to charity.^6^ If so, they could still donate money to charity (one could even use policies which promote such donations). Hence, there are ways to mitigate the effects on the benefits of philanthropy even if limitarian policies lead to leveling down, and assuming that the benevolent rich are fair agents of justice in the first place.

Furthermore, there is a deeper worry with extreme wealth and vast wealth inequalities that Volacu and Dumitru do not address.^7^ Whether or not the rich are fair and just, extreme wealth creates power outside the political sphere as well and, in that way, creates pernicious domination (see e.g. Cohen 1995, pp. 34–37; Anderson 1990, pp. 192–201). Wealth can be used to encroach on the liberty of others, thereby exercising enormous power over their every-day life. In real life, under the current distribution of wealth, the purchasing power of the very wealthy ranges over schools, land, houses, factories, companies, hospitals, etc. and through this they gain enormous power over the lives people are living. Hence, the mere fact that they are wealthy is problematic due to domination in non-political areas of life. This means that possible benefits of allowing the rich to meet unmet urgent needs and establish other kinds of goods must be weighed against worries about power and pernicious domination.

## The Efficacy Objection

Recall that the Democratic Argument holds that to the extent that limitarianism limits excessive wealth, limitarianism is pro tanto justified as a means to promote political equality. But what if there is no direct relation between limiting wealth and furthering political equality? What if, as the Efficacy Objection holds, limitarian policies can “in fact not [guarantee] to genuinely safeguard political equality”? (Volacu and Dumitru 2018, p. 9) Let us take a closer look at this Efficacy Objection. According to Volacu and Dumitru, the reason why limitarian policies fail to establish political equality is that people can choose to spend income below the limitarian threshold in different ways. Take, for example, a democratic society in which no one has less than €100.000, in which everyone has precisely this amount of money, and in which it is impossible to have more than €100.000. Person A may prefer to spend her money on an expensive holiday, whereas person B may choose to spend it on influencing politics. If person B is not alone but operates as a member of a group of politically engaged peers, it is likely that political equality cannot be guaranteed by strong limitarianism. This is because in the end person B and her peers will have a much greater capacity to influence politics than person A. Volacu and Dumitru therefore argue that other institutional measures than strong limitarianism must be adopted to ensure political equality. But does the Efficacy Objection hold?

According to the Democratic Argument, political equality requires that nobody should have more or less political influence because of their (lack of) wealth.^8^ The Efficacy Objection, then, holds that limitarian policies cannot prevent some people from having more or less political influence just because they are richer. However, limitarian policies need not do all the heavy lifting. They are a *partial* means to promote political equality. And so the Efficacy Objection, it seems to me, raises two questions: First, are limitarian policies *effective* as a partial means to promote political equality? Second, are limitarian policies, even as a partial means, actually *needed* to establish political equality? In what follows, I will give four arguments as to why limitarian policies are both effective and needed. Such policies address issues which other policies cannot address, and they promote political equality at least partially.

First, financial capital, i.e. wealth, tends to attract nonfinancial capital, such as knowledge and opportunities (‘social capital’), and behavioral norms and dispositions (‘cultural capital’) (cf. Halliday 2018, p. 107). The differences in access to nonfinancial capital that come with being a member of a specific social group translate into differences in the capacity to influence politics. Over time, this leads to what Halliday labels ‘economic segregation’, which “occurs when an individual’s life prospects, and/or social status, depend on his or her group membership—specifically, membership of a group that possess greater wealth than other groups” (Halliday 2018, p. 1). Insofar as, for example, inheritance plays an important role in maintaining economic segregation over time, tackling inequalities which result from inheritance can tackle economic inequality. It is precisely these kinds of policies which policy limitarianism proposes. And insofar as the distribution of financial and nonfinancial capital is a proxy for the distribution of the capacity to influence politics, such policies promote political equality.

Second, people tend to be cognitively biased towards their own well-being, conception of the good and the role they play in their financial success (cf. Christiano 2008, pp. 58–60; Halliday 2018, pp. 113–117). There is insightful empirical evidence to support this claim. Research shows that people tend to have a self-serving bias, which is the tendency to perceive of themselves favorably. This self-serving bias contributes to polarization on the views about predistribution and redistribution that people hold (Deffains et al. 2016). The highly unequal distribution of wealth in Western societies reinforces the impact of cognitive bias on political equality (Alvaredo et al. 2018). People are biased against seeing the illegitimacy of being rich and the political influence that comes with it, and the illegitimacy of others having much less of both.^9^ This poses a problem for those caring about political equality. As Anderson puts it, the “self-serving bias is pervasive [but] has asymmetrical effects across social positions. It afflicts the powerful more than the powerless, and those with unaccountable power most of all. As Dewey observed: It is difficult for a person in a place of authoritative power to avoid supposing that what he wants is right as long as he has power to enforce his demand” (Anderson 2014, p. 7). Insofar as wealth is a proxy for political influence, one must care about riches not for the sake of reducing inequality but for the sake of equalizing political influence.

Third, what limitarian policies establish is that the opportunity costs for spending money on influencing politics are more equal for everyone, which furthers political equality. This holds even if limitarian policies do not rule out the possibility that some people are able or willing to spend more money on influencing politics than others, for example by pooling their resources. Limitarian policies cannot prevent some people from influencing politics on the grounds that they are richer, but in a society in which no-one or fewer people are excessively rich it is *more likely* that people will not have more influence just because they are richer. And, again, limitarian policies are a partial means that can supplement other institutional and constitutional changes to promote political equality.

Fourth, and finally, reducing inequalities in wealth leads to less discrepancies between the interests of people in society in certain domains of life. Limitarian policies protect people’s interest in political equality by making people’s interests more similar, such as their interest in a welfare state. Even if some people choose to spend more money on influencing politics than others, promoting their own interests will more likely entail furthering other’s interests as well.^10^

## Conclusion

In this paper, I have defended policy limitarianism as a non-ideal and partial view on how to instantiate and promote political equality. I have argued that policy limitarianism can disarm the Incentive Objection and the Efficacy Objection that Volacu and Dumitru raise against Robeyns’ Democratic Argument for limitarianism. I have rejected the Incentive Objection on the grounds that there are different limitarian policies from the ones discussed by Volacu and Dumitru and which escape the problem of reduced productivity. I have rejected the Efficacy Objection because limitarian policies are a partial but highly valuable step towards establishing political equality, and that they can and should complement or be complemented by other strategies.

Much more needs to be said to spell out the implications of the policy limitarian view that I have developed in this paper, and to assess its normative foundation and empirical viability. The underlying aim of this paper, however, has been more modest, namely to show that policy limitarianism deserves more credit as a means to promote political equality than may seem from Volacu and Dumitru’s assessment of the Democratic Argument.

## Footnotes

1 To clarify, limitarianism is a partial view about distributive justice because it holds that limiting wealth is necessary but not sufficient for a fair distribution. As a non-ideal doctrine, limitarianism starts “from the distribution of the possession of income and wealth as it is, rather than asking what a just distribution would be in a world with strong idealized properties” (Robeyns 2017, p. 2).

2 The distinction between strong limitarianism and weak limitarianism is introduced by Volacu and Dumitru but is not used by Robeyns. Weak limitarianism, Volacu and Dumitru argue, is called for by Robeyns’ second argument for limitarianism, namely the Argument from Unmet Urgent Needs (cf. Robeyns 2017, pp. 10–14). Volacu and Dumitru (2018, pp. 10–14) adopt a specific version of this argument combined with a responsibility-catering interpretation of weak limitarianism.

3 A different way to state this argument is to say that Volacu and Dumitru’s so-called “institutional measures view” is not an alternative to limitarianism, but that limitarianism explores specific institutional measures (cf. Volacu and Dumitru 2018, pp. 7–8).

4 I thank an anonymous reviewer for this suggestion.

5 For a more elaborate description of the Rignano scheme and some reflections on its relation to incentives, see Halliday (2018), pp. 58–72, 194–197.

6 Volacu and Dumitru (2018, p. 9–10) concede as much when they argue that limitarian policies are ineffective because they do not prevent the pooling of wealth by a few to gain political influence. On this point, see Section 4.

7 I thank an anonymous reviewer for this argument.

8 This requirement does allow certain inequalities in political influence, for example because of efficiency gains or because some people just have better ideas than others (cf. Christiano 2005).

9 I thank an anonymous reviewer for stressing this point.

10 An example of this would be a maximum wage that is linked to a specific wage ratio, see e.g. Pizzigati (2018, ch. 2).
